# The effect of regular walks on various health aspects in older people with dementia: protocol of a randomized-controlled trial

**DOI:** 10.1186/1471-2318-11-38

**Published:** 2011-08-09

**Authors:** Karin M Volkers, Erik JA Scherder

**Affiliations:** 1 Department of clinical neuropsychology, VU university Amsterdam, van der Boechorststraat 1, 1081 BT Amsterdam, the Netherlands; 2 Institute for human movement sciences, university of Groningen, A. Deusinglaan 1, 9713 AV Groningen, the Netherlands

## Abstract

**Background:**

Physical activity has proven to be beneficial for physical functioning, cognition, depression, anxiety, rest-activity rhythm, quality of life (QoL), activities of daily living (ADL) and pain in older people. The aim of this study is to investigate the effect of walking regularly on physical functioning, the progressive cognitive decline, level of depression, anxiety, rest-activity rhythm, QoL, ADL and pain in older people with dementia.

**Methods/design:**

This study is a longitudinal randomized controlled, single blind study. Ambulatory older people with dementia, who are regular visitors of daily care or living in a home for the elderly or nursing home in the Netherlands, will be randomly allocated to the experimental or control condition. Participants of the experimental group make supervised walks of 30 minutes a day, 5 days a week, as part of their daily nursing care. Participants of the control group will come together three times a week for tea or other sedentary activities to control for possible positive effects of social interaction. All dependent variables will be assessed at baseline and after 6 weeks, and 3, 6, 9, 12 and 18 months of intervention.

The dependent variables include neuropsychological tests to assess cognition, physical tests to determine physical functioning, questionnaires to assess ADL, QoL, level of depression and anxiety, actigraphy to assess rest-activity rhythm and pain scales to determine pain levels. Potential moderating variables at baseline are: socio-demographic characteristics, body mass index, subtype of dementia, apolipoprotein E (ApoE) genotype, medication use and comorbidities.

**Discussion:**

This study evaluates the effect of regular walking as a treatment for older people with dementia. The strength of this study is that 1) it has a longitudinal design with multiple repeated measurements, 2) we assess many different health aspects, 3) the intervention is not performed by research staff, but by nursing staff which enables it to become a routine in usual care. Possible limitations of the study are that 1) only active minded institutions are willing to participate creating a selection bias, 2) the drop-out rate will be high in this population, 3) not all participants will be able to perform/understand all tests.

**Trial registration:**

NTR1482

## Background

Aging may coincide with a decline in the level of physical activity [1]. This age-related decline in physical activity is often related to difficulties in physical performances, e.g. walking and getting out of a chair [2,3]. There is ample evidence that in cognitively intact older people living in the community or in long-term care, physical performance can be enhanced by physical activity interventions [4-10]. It is suggested that the intensity of the intervention is positively related to its effectiveness, i.e. a higher intensity produces a higher effect [11]. Nursing home residents are sometimes considered as too frail or too cognitively impaired to benefit from physical activity interventions, but cognitively impaired people seem to benefit from physical activity interventions just as much as cognitively intact people [7]. Furthermore, it has been suggested that frail dependent people only benefit from frequent individualised interventions, while less frail people already respond positively to traditional group interventions [12]. Other studies, including residents with and without cognitive impairment [7,10] or only people with cognitive impairment [11,13-16], confirmed that all residents can improve physical performances through physical activity, but evidence of high-quality studies is still limited or inconclusive.

The risk of dementia might be reduced by physical activity due to its positive effect on cognition [4,17-21], especially on executive functions (EF) [18,21]. When people already show a cognitive decline or suffer from dementia, which highly affects cognition due to neuropathology [22], results on the effects of exercise on cognition are equivocal [13,14]. An explanation for not finding a beneficial cognitive effect was that the interventions mainly consisted of strength-, balance- and/or flexibility-based exercises [14]. A recent meta-analysis indicated that the evidence is not sufficient for firm conclusions about the effectiveness of physical activity interventions on cognition in dementia [23]. The evidence that is available is promising since exercise has a large effect on cognition (Cohen's d = 1.31) and was observed as being significantly different from the control group (moderate effect: Cohen's d = 0.56 and 0.57) [16,24]. More research is warranted, because the available studies differ in exercise protocols, durations and outcome measures [14,23].

One of the risk factors for dementia is an increased level of anxiety and/or depression [25]. In healthy older people physical activity interventions can increase mental health, e.g. self-esteem, happiness and mood [4,26]. Looking explicitly at depression and anxiety, physical activity interventions can reduce the depressive symptoms within 3 months in older people with a depression [27] or anxiety after 3 weeks in people with a chronic physical condition, e.g. fibromyalgia [28]. Long-term effects of exercise on depressive symptoms and anxiety remain to be demonstrated in clinical trials [4,27]. Even though physical activity as a treatment for depression may be as effective as medication, it is currently under-used for this disorder despite having many additional health benefits, for example a reduced risk of heart disease, stroke, high blood pressure, some cancers, type 2 diabetes, osteoporosis and obesity [27]. This makes a physical activity treatment appropriate for older people with a combination of physical and mental health problems such as dementia [27]. In people with dementia evidence of helping with depression is limited [11,23], but the results of available studies are promising [13,14].

Together with a rising level of depression and cognitive impairment, the prevalence and frequency of sleep disorders in long term care residents is increasing [29]. More than half of the residents suffer from some kind of sleep disorder [29]. Night-time wakefulness can lead to increased daytime sleeping and vice versa [30]. Sleep disorders and a lack of activities negatively influence each other [29]. Long-term care residents spend extended periods of time in bed and are sedentary during the daytime, which contributes to abnormal circadian rhythms, i.e. rest-activity rhythm [30]. In older persons with or without dementia, a physical activity intervention seems to be effective [24,31-34], especially in people with poor sleep at baseline [33]. These studies, however, are scarce since the majority of physical activity intervention studies are multi-dimensional, i.e. physical activity is combined with bright light or a decrease in noise at night [24,30,31,33]. Such a combination hampers the understanding of which intervention is (most) effective [35]. A disturbance in the rest-activity rhythm is one of the prominent clinical symptoms in people with dementia which should be recognized and enhanced, since it has a high impact on the quality of life (QoL) [31].

QoL is determined by many aspects in life, e.g. quality and quantity of sleep [36], cognitive functioning [36] and level of depressive symptoms [37]. Exercise programs can slow down the decline in QoL in older community dwelling people [38] and in a combined resident group of people with and without dementia [39]. However, for a group including only people with dementia the evidence for an effect on QoL due to physical activity is limited [11,13,23].

QoL is also affected when people with dementia lose the ability to cope with the physical activities of daily life (ADL), e.g. eating, bathing, using the toilet, dressing, walking and continence [40]. Due to participation in a physical activity intervention, a combined resident group of people with and without dementia can prevent or reduce a decline in ADL compared to a control group [12,39]. In groups with only older people with dementia, there is some evidence that physical activity can improve the ability to cope with ADL [16,41], but there were not sufficient studies for a meta-analysis to conclude whether or not physical activity could be effective for ADL performance in people with dementia [13,23].

The risk to decline in ADL performance increases by approximately 20% for each (additional) painful part of the body, i.e. the neck, back, hands, hips, knees or feet [42]. In older people pain is often caused by the musculoskeletal condition of the joints [43]. In cognitively intact older people a dose-response relationship exists between the level of physical activity and pain; a higher physical activity level was related to less stiff and painful joints [43] and people experienced less pain during a cancer treatment when they performed more aerobic exercises, e.g. walking [44]. In addition, there is more evidence that pain can be reduced by stretching activities [45] or resistance training [46] and specifically (low) back pain by resistance, agility or stretching activities [46]. In contrast, a moderate-intensity endurance and strengthening program had no effect on pain in healthy elderly [45] and neither had a 12 weeks walking program in older people with dementia [47]. No other studies including people with dementia examined the effect of physical activity on pain even though this is necessary since pain is often under-diagnosed and under-treated in people with dementia [48,49].

Studies related to older people with (or without) dementia have not been able to reach a consensus on the types and intensity of the exercise, nor the frequency and duration of the intervention to be most effective and efficient [14]. This is due to the variety in outcome measures; one outcome measure can respond faster to a physical activity intervention than another outcome measure [14]. The effect on outcome measures is also dependent on the age of participants, the cognitive impairment and frailty level [14]. However, in both frail and non-frail older adults regular exercise is the only therapy found to consistently improve health aspects, e.g. physical function, cognitive performance and mood [4]. Most meta-analyses and reviews have concluded that longitudinal randomized controlled trials (RCT) are of vital importance [4,9,10,12-14,18-20,50,51]. Especially people with more severe cognitive impairment should be included in future research [14]. As mentioned above, physical activity has proven to be beneficial to improve or slow down many health aspects in older people with dementia. The aim of the present study is therefore to investigate longitudinally the effect of regular walking on physical functioning, cognition, level of depression and anxiety, rest-activity rhythm, QoL, ADL and pain in older people with dementia.

## Methods/design

### Participants

Participants are older mobile people with dementia who are visiting day care or living in a home for the elderly or nursing home in the Netherlands. Inclusion criteria are 1) a diagnosis of dementia or presence of cognitive impairment that is reported in the medical status and 2) ambulatory with or without walking aid (walker or cane). Exclusion criteria are the presence of personality disorders, cerebral traumata, hydrocephalus, neoplasm, disturbances of consciousness and focal brain disorders.

### Study design and randomization

This study is a RCT. Participants will be randomly divided into the intervention or control group. In order to preclude that the effects arise only from the intensified social contacts during intervention, the control group within a residency will come together three times a week for tea or other sedentary activities, e.g. watching television. The Medical Ethical Committee of VU university medical center approved the study. Oral and written informed consent will be obtained from all participants or their relevant relatives prior to their enrolment.

### Intervention

The intervention consist of a daily 30 minute walk, 5 times a week under supervision of an assistant, e.g. medical staff, volunteers or family members. Depending on the participant, the assistant, the living area and the weather, the intervention can take place in the morning or afternoon, inside or outside, during midweek and/or weekends. Each day the participant has walked will be carefully noted, together with the time of day, duration and whether it was inside or outside.

### Sample size

Sample size calculation was performed using the statistical power analysis program G*Power 3.1 [52] which has the possibility to perform power analyses for a repeated measures design with a between group variable. Based on two meta-analyses studying the effect of physical activity on cognition [16,24], the estimated effect size in this study is expected to be moderate to large (Cohens f = 0.32). Using a type 1 error of 0.05, a power of 0.80, seven repeated measurements and 2 groups, a sample size of 70 in each group is required. Taking into account an attrition rate of 25%, 175 participants should be included in this study.

### Recruitment

Participants for this study will be recruited via medical staff of aged care facilities. First, the medical staff will be informed about the goal and procedure of the study. Secondly, possible participants will be selected within subunits of the institutions by a team, including the researcher, medical staff and nurses. Third, an information letter with informed consent will be sent to the legal representatives of the participants, together with an invitation to attend an oral presentation. Fourth, once written consent is received, subunits make a schedule for assistants to accompany the regular walks of participants in the intervention group.

### Procedure

The outcome variables are measured at baseline (pre-treatment: T0), after 6 weeks of intervention (post-treatment: T1) and after 3 (T2), 6 (T3), 9 (T4), 12 (T5) and 18 (T6) months of intervention. Trained experimenters, blinded to the intervention assignment, will administer the neuropsychological tests, physical tests and level of depression, anxiety and pain. The nursing staff fills in questionnaires to measure QoL, ADL and define the stage of dementia. The other variables are objective measurements.

### Characteristics

Characteristics include *age*, *gender*, *subtype of dementia, type of living situation*, i.e. independently in society, in a home for the elderly or in a nursing home as well as the following aspects:

#### Highest education level

The highest education level will be determined on a seven-point scale with 1 = less than elementary school, 2 = 6 grades of elementary school, 3 = 7 or 8 grades of elementary school, 4 = 3 years of lower general secondary education, 5 = 4 years of lower general secondary education, 6 = pre-university education and higher vocational education, 7 = university and technical college [53].

#### Body mass index (BMI)

BMI is calculated as weight in kilograms divided by height in meters squared.

#### Apolipoprotein E (ApoE) genotype

ApoE type 4 allele (ApoE4) plays a major role in cerebral perfusion and metabolism and is a known risk factor for late onset Alzheimer's disease (AD) [54]. In addition, ApoE4 carriers show different effects on cognition as a result of some therapy than non-carriers [55,56]. In view of this possible moderating effect on treatment outcome, ApoE genotype of participants will be determined. Buccal swabs will be taken by making use of Catch-all™ collection swabs (Epicentre, Madison, Wisc., USA). First, participants must rinse their mouth thoroughly with water. Then two swabs are taken from each participant, will be left to dry for approximately 30 minutes and are frozen until DNA will be released from the swab by a rapid lysis technique [57] and the nucleotide sequence will be determined [58].

#### Stage of dementia

To assess the stage of dementia of participants, the Dutch version of the global deterioration scale was used [59]. This classification scale indicates the severity of the dementia from pre-dementia stages (1 to 3) to profound (stage 7).

#### Comorbidity

Comorbid conditions are extracted from the medical status and categorized based on the Dutch translation of the Long-Term Care Facility Resident Assessment Instrument (RAI), section I. This section (disease diagnoses) includes the following categories: 1) *endocrine/metabolic/nutritional*, i.e. diabetes mellitus, hyperthyroidism and hypothyroidism, 2) *heart/circulation*, i.e. arteriosclerotic heart disease, cardiac dysrhythmia, congestive heart failure, deep vein thrombosis, hypertension, hypotension, peripheral vascular disease and other cardiovascular disease, 3) *musculoskeletal*, i.e. arthritis, hip fracture, missing limb (e.g. amputation), osteoporosis and pathological bone fracture, 4) *neurological*, i.e. AD, aphasia, cerebral palsy, cerebrovascular accident, dementia other than AD, hemiplegic/hemi paresis, paraplegia, multiple sclerosis, Parkinson's disease, seizure disorder, transient ischemia attack, traumatic brain injury and quadriplegia, 5) *sensory*, i.e. cataracts, diabetic retinopathy, glaucoma and macular degeneration, 6) *psychiatric/mood*, i.e. anxiety disorder, depression, manic depression (bipolar disorder) and schizophrenia, 7) *pulmonary*, i.e. asthma and emphysema/chronic obstructive pulmonary disease, 8) *other*, i.e. allergies, anaemia, cancer and renal failure.

#### Medication

Medication use is coded according to the Dutch Pharmacotherapeutic Compass and is ranged by the following groups: 1) sedatives, 2) antipsychotics, 3) antidepressants, 4) pychotropics (central nervous system (CNS)), 5) neurological (CNS), 6) anaesthetics and muscle relaxing, 7) blood, 8) cardiovascular, 9) gastrointestinal tract, 10) respiratory tract, 11) kidneys and urinary tract, 12) genital tract, 13) dermatology, 14) otolaryngology, 15) ophthalmologic, 16) infectious diseases, 17) hormones and bone metabolism, 18) corticosteroids nonsteroidal anti-inflammatory drugs (NSAID), 19) corticosteroids nonNSAID, 20) analgesics, antirheumatic drugs and gout agents, 21) vitamins and minerals, 22) malignancies, 23) infectious diseases, 24) various preparations, 25) dentistry, 26) opioids.

### Assessment of physical functioning

For all tests of physical functioning, participants wear their regular footwear and are permitted to use their regular walking aid. As encouragement has been shown to improve performance [60], standardized encouragements will be provided regularly. All tasks are explained and demonstrated to the participants before testing. No practice trials are included. Participants perform the tests under continuous supervision of the researcher to prevent the participants forgetting what they have to do during the tests.

#### Blood pressure

Systolic blood pressure (SBP) and diastolic blood pressure (DBP) are measured on the left arm in millimeters of mercury (mmHg) with an ambulatory blood pressure monitoring device (model 90207, SpaceLabs Medical Inc., Redmond, Washington, USA) while participants are sitting quietly in a chair for at least 5 minutes [61]. This device is automated, lightweight, calibrated and validated [62]. Observers do not wear a white-coat to prevent higher blood pressure measurements due to a white-coat effect and multiple cuff bladders are used to measure blood pressure with a cuff bladder that encircles at least 80% of the arm [61]. In particular, blood pressure of participants with hypertension, i.e. a higher SBP than 160 mmHg and/or a higher DBP than 100 mmHg is expected to decrease during treatment [63]. The SBP and DBP reduction represents the clinical effectiveness of the treatment [64].

#### Six minute walk test (6MWT)

The 6MWT can be used reliably in the assessment of functional endurance ambulation in persons with acquired brain injury [65]. During the performance of the 6MWT, participants are instructed to cover as much distance as possible during 6 minutes with the opportunity to stop and rest if necessary [66]. Participants have to walk around a pre-measured, unobstructed 10 by 1 meter rectangular circuit having semi-circular ends with 0.5 meter radii marked out with plastic cones to prevent participants having to walk at sharp angles. One full round covers 26.3 meters walking. The total walking distance by each participant will be measured to the nearest meter. To estimate the effort of participants during the 6MWT the increase in heart rate will be determined (see heart rate) [67].

#### Heart rate

Heart rate at rest is simultaneously measured with blood pressure. This blood pressure device (see blood pressure) also measures heart rate in beats per minute. In addition, heart rate is measured with a pulse oximeter (CMS 60C TFT color Pulse oximeter) before and directly after the 6MWT for 2 minutes. The faster the heart rate recovers within 1 minute after the 6MWT the healthier the heart; a slower heart rate recovery is associated with more severe coronary artery disease [68].

#### Oxygen saturation

Arterial oxygen saturation is measured before and directly after the 6MWT to determine dyspnea [69]. This is measured with the same pulse oximeter as mentioned above, simultaneously with heart rate. Oxygen saturation is not measured during the 6MWT due to motion artefact which results in unacceptably high failure rates of the devices [66].

#### Ten meter timed walk

Participants are requested to walk 10 meters at their own regular pace between 4 small traffic cones which are placed in the corners of a 10 by 1 meter rectangle. Their walk is filmed from behind by a digital video camera (Panasonic NV-GS330, Matsushita Electric Industrial Co., Ltd. Osaka, Japan) standing at least 8 meters behind the start on an 24.25 inch tripod (Vanguard MK-4). Time to walk 10 meters is measured by hand with a stopwatch to the nearest of 1/10 of a second and by video-analysis. By video-analysis also walking speed measured over 6 meters, i.e. without the 2 meters at start and 2 meters at finish to exclude starting hesitation and the slowing down at finish, step frequency, base width and step length will be analysed.

#### Figure of eight

The figure of eight is an applicable and reliable dynamic functional balance measure of mobility for people with various degrees of physical disability [70,71] and geriatric patients [72]. The figure of eight test requires continuous turning during gait with an emphasis on accuracy (avoid oversteps), speed (timed task) and switching of motor patterns during the cross-over from the clockwise to the counter-clockwise loop. Participants are timed while walking in a figure-8 trajectory. The figure-8 trajectory is marked with white paint on a dark green rubber carpet, each loop having an outer diameter of 165 centimeters (cm) and a step width of 15 cm. The time to walk two complete eight figures is measured with a stopwatch. The onset time is based on the first detectable movement of the participant following a "Go!" command from the observer. Any step taken outside the white line is noted. The fastest attempt of two trials is recorded together with the corresponding oversteps. Speed (m/s) is 19.792 meter divided by the time in seconds.

#### Timed up and go (TUG)

The TUG is a reliable and valid test for quantifying functional mobility that may also be useful in following clinical change over time [73]. To complete the TUG, participants are requested to rise from a standard chair (48 cm height, horizontal seat and armrests), walk 3 meters, turn around and return to a fully seated position in the chair again [74]. Each participant has two trials and the average time in seconds is the outcome of the TUG. Scores under 10 seconds are associated with individuals who are functionally independent in the frail elderly population [74].

#### Sit to stand (STS)

The STS is normally a reliable and valid indicator of lower body strength in adults over the age of 60 years [75]. However, in this study participants are allowed to use upper limbs to rise from the chair to test their rising performance that is closest to the clinical setting and to reduce a floor effect; a high percentage of older dependent elderly cannot rise from a chair with the arms crossed in front of the chest [76,77]. Participants are instructed to stand up and sit down in a standard chair as many times as possible within 30 seconds. The STS score is formed by the total number of performances with a sit-stand-sit performance counting as 1. Ending in a standing position is counted by a 0.5 point.

#### Frailty and injuries: cooperative studies of intervention techniques (FICSIT-4)

The FICSIT-4 is a test to measure static balance [78]. The participants have to maintain balance in 4 positions with increasing difficulty. Each position is demonstrated first and support is offered while participants position their feet. When participants are ready, the support will be released and timing begins. The timing stops when participants move their feet or grasp the researcher for support, or when 10 seconds have elapsed. Only when one stand is performed 10 seconds, the next, more difficult stand is performed. The first stand is with the feet together in parallel (side-by-side) position. Second is the semi-tandem position; the heel of one foot is placed to the side of the first toe of the other foot. The participant can choose which foot to place forward. Third is a tandem position; the heel of one foot directly in front of the toes of the other foot. The final stand is standing on one leg. The total summed seconds of all stands is the outcome score.

### Assessment of cognition

Cognition will be assessed by the following neuropsychological tests:

#### Mini-Mental State Examination (MMSE)

The MMSE measures the global level of cognitive functioning [79]. Globally, a score between 25 to 30 indicates no dementia, between 15-24 mild dementia, between 5-14 moderate to severe dementia and between 0 to 4 profound dementia [80].

#### Eight words test

The eight words test is a list learning test for people with memory problems [81]. In this test the examiner reads out eight words in a row, which is repeated five times. Every time the participant is asked to recall as many words as possible. The first outcome measure is the total number of correctly recalled words after the five trials (*immediate recall score*, maximal score = 40). After an interval of approximately 15 minutes the participant is asked to recall as many words as possible (*delayed recall score*, maximal score = 8). Subsequently, the examiner reads aloud 16 words among which 8 words presented before and 8 new words. The participant is asked to recognize the words from the list presented before (*recognition score*, maximal score = 16).

#### Rule shift cards

The rule shift cards is a subtest of the Behavioural Assessment of the dysexecutive Syndrome (BADS) [82]. This subtest purports mental flexibility and is one of the best qualifiers to discriminate people with perseverative tendency, i.e. an executive dysfunction, from healthy people [83]. Participants have to respond to stimuli (red or black playing cards) according to one of two rules that are presented consecutively. Performance is scored according to how successfully the respondent shifts from applying the first to the second rule [82].

#### Key search

The key search test is also a subtest of the BADS [82] and is used to measure how well the participant is able to prepare an efficient plan of action in the context of a routine event [83]. The patient is asked to imagine that a 100-mm square on an A4 size paper is a large field, in which they have lost their keys. They are asked to draw a line, beginning at a black dot, 50 mm below the square, to show the strategy they would use to search the field, to make absolutely certain that they would find their keys. The test evaluates a person's ability to monitor and evaluate their own performance, taking into account factors that are not explicitly stated in the instructions. Their search strategy score is based on a number of criteria, such as efficiency and effectiveness. The best strategy score is 15 and the worst score is 2.

#### Digit span (forward and backward)

The digit span is a subtest from the Wechsler Memory Scale-Revised (WMS-R) [84]. In the digit span forward, increasingly long sequences of random numbers are orally presented at a rate of one digit per second to the participants, who have to repeat the sequence immediately after oral representation. In the digit span backward, participants have to repeat the sequence in reverse order. To do this, participants perform extra mental operations on the information that is being held in short term memory. While the backward condition is often hypothesized as tapping more into EF than the forward condition which should measure more short term memory, research has failed to demonstrate this result [85,86]. These studies suggest that both conditions tap into EF. Each condition ends when a participant fails to recall at least two strings of the same length or repeated an eight-digit sequence correctly. The minimal score for both conditions is 0 and the best score is 21.

#### Face recognition

Face recognition is a subtest from the Rivermead Behavioral Memory Test (RBMT) [87] and measures visual, nonverbal long term memory. Two versions (C+D) are combined to prevent a ceiling effect. In this test the participant is shown 10 cards with faces one at a time for 5 seconds. After a short interval of approximately 2 minutes, the participant is shown 20 cards, including 10 shown before and 10 cards with new faces. The participant has to recognise whether the card was shown before or not. The outcome measure is the number of faces correctly recognized minus the number of faces incorrectly recognized. The worst score is -20 and the best score is +20.

#### Picture recognition

Picture recognition is also a subtest from the RBMT [87], which measures visual, verbal long term memory. Two versions (C+D) are combined to prevent a ceiling effect. The participant is shown each of the 20 cards with drawings of objects for 5 seconds. With each card, the participant is requested to name the object on the card. After a short interval of approximately 2 minutes, the participant is shown 40 cards, including 20 shown before and 20 cards with new objects. The participant has to recognise whether the card was shown before or not. The outcome measure is the number of objects correctly recognized minus the objects that were incorrectly recognized. The lowest score is -40 and the maximal score is +40.

#### Category fluency test

The category fluency test is a verbal fluency test which can be used to evaluate EF [88,89]. The participant is asked to name as many examples of a given category as possible, within 1 minute. This requires a strategic search mechanism to retrieve information from semantic memory [90]. This study uses the category 'animals' and 'professions' [91]. The outcome measure is the total number of animals and professions produced.

#### Visual memory span (forward and backward)

The visual memory span is a subtest of the WMS-R [84]. The visual memory span stimuli consist of squares printed on a two dimensional card and requires the participant to repeat a number of tapping sequences that becomes longer with each trial. The visual memory span contains a forward and a backward sequence, similar to the digit span test. It was initially added to the WMS-R as a visual analogue digit span test. The forward condition is used as a measurement of attention and immediate visual memory, the backward condition is used as a measure of attention and visual working memory [84]. Scores range from 0 (worst) to 14 (best) in the forward condition and from 0 (worst) to 12 (best) in the backward condition.

#### Picture completion

Picture completion is a subtest from the Groninger Intelligence Test (GIT) [92]. The GIT is a test of general intelligence that is used in the Netherlands for purposes comparable to the Wechsler Adult Intelligence Scale (WAIS). The picture completion subtest measures visual perception, specifically, alertness to visual detail [93]. Available literature suggests that this subtest may have utility as a measure of suboptimal effort, especially in less-educated participants and is a moderately effective measure of response bias [93]. Figures are not completely drawn and participants are instructed to describe the missing parts of pictured objects. The figures increase in difficulty. After 20 figures or after 5 false descriptions in a row, the subtest is finished. The best score is 20, representing 1 point for every correct description.

#### Stroop task

The version of the Stroop task that is commonly used in the Netherlands [94] consists of three subtasks which the participant performs as quickly as possible; each test has a 45 second time limit. In the first subtask, participants are presented with four color words, i.e. red, green, blue and yellow, printed in black ink in ten rows with ten names each. The participant's task is to read as many color names in the right order. Second, the participant is presented with a similar paper, but this time with solid color patches (red, green, blue and yellow) that have to be named in the right order. In the final subtask the names of the colors are written in a different color ink than the meaning of the word (i.e. the word 'blue' written in red ink) and the participants again need to name the color of the ink, thus suppressing reading the word, which is a highly automatic reaction. This test has been found to correlate moderately well with other tests of response inhibition [95], which is an EF task [96]. The score on each card is the total correct mentioned colors within 45 seconds. The final score is the score on card 2 minus the score on card 3. A lower final score indicates a better performance of inhibition.

#### Digit symbol substitution test (DSST)

The DSST is a subtest of the WAIS-Revised [84] and has been widely used as a measure of general information processing speed in studies of cognitive aging [97]. Test scores correlate with general intelligence, cognitive impairment, chronological age and activation in the frontal regions [98-101]. Participants are presented with a rectangular grid of numbers. For each of these numbers, participants are instructed to substitute the appropriate symbol according to a code that appears at the top of the page. The DSST score is recorded as the number of correct symbols drawn in 2 minutes.

### Assessment of depression level and anxiety

The level of depression and anxiety will be assessed by the following questionnaires:

#### Geriatric depression scale (GDS)

The Dutch version [102] of the GDS is a 30-item questionnaire used to measure general mood [103]. The GDS is a reliable and valid self-rating depression screening scale for elderly populations [104]. The GDS questions are answered by 'yes' or 'no' depending on which response is most appropriate at the time of measurement, with 0 or 1 point for each answer. Higher scores indicate a higher level of depression.

#### Symptoms checklist 90 (SCL-90)

Two subscales from the Dutch version [105] of the SCL-90, a 90-item self-report symptom inventory designed to reflect patterns of current psychological symptoms, will be used to measure depression and anxiety [106,107]. The depression and anxiety subscale includes 15 and 10 items respectively. Each item is rated on a 5-point likert scale, from 1 (not at all) to 5 (extremely). A higher score indicates more symptoms of depression or anxiety.

### Assessment of rest-activity rhythm

Rest-activity data are collected by the use of an Actiwatch activity monitor (Cambridge Neurotechnology Ltd., Cambridge, Great Britain). Actiwatches are small activity monitors worn on the dominant wrist for several days. Three variables below are analysed.

#### Interdaily stability (IS)

The IS serves as a measure to which extent the activity patterns of all included 24 hour periods resemble each other. IS is calculated as the ratio between the variance of the average 24 hours pattern around the mean and the overall variance [108]. Higher values indicate a more stable rhythm between days.

#### Intradaily variability (IV)

The IV quantifies how well the continuity of an arousal state (sleep/activity) is. Normal rest-activity patterns will show every 24 hours one major active period (day) and one major resting period (night) and therefore show a low IV. IV is calculated as the ratio of the mean squares of the difference between successive hours (first derivative) and the mean squares around the grand mean (overall variance) [108]. Lower values indicate a better rest-activity pattern.

#### Relative amplitude (RA)

The RA measures the relative difference in the 10 most active consecutive hours (M10) and the uninterrupted least active 5 hours period (L5) within a 24 hours cycle. RA is calculated as the difference between the means of M10 and L5 in the average 24 hours pattern [108]. Because it is a relative measure, variance resulting from differences in sensitivity of actigraphs is reduced. Higher values indicate a larger difference between daytime activity and night time rest and therefore a better rhythm.

### Assessment of QoL

#### Qualidem

The Qualidem is a reliable and valid 40-item questionnaire designed to determine QoL in institutionalized residents with dementia [109-111]. The questionnaire includes indicative and contra-indicative items that can be divided into 9 homogeneous subscales: 1) care relationship, 2) positive affect, 3) negative affect, 4) restless tense behaviour, 5) positive self-image, 6) social relations, 7) social isolation, 8) feeling at home, 9) having something to do. The items are printed in random order, so that items of a subscale are spread within the questionnaire. Each item has 4 possible responses; 'never', 'rarely', 'sometimes' and 'often'. Each response is scored with 0 to 3 points, with higher scores indicating higher QoL. The questionnaire is completed by the nursing staff and varies from the highest QoL (score 120) to the lowest QoL (score 0).

### Assessment of ADL

#### Katz index

The Katz index is a 6-item measure of basic human activities of daily living: bathing, dressing, toileting, transfer, continence and feeding [112]. The scale is completed by the nursing staff and varies from complete independency (score 6) to maximum dependency (score 18).

### Assessment of pain

Pain will be assessed by the following pain scales:

#### Coloured analogue scale (CAS)

The CAS will be used to determine both pain intensity and unpleasantness, i.e. affect [113]. The CAS includes 2 pain thermometers which are white at the bottom and red at the top to measure both aspects of pain. It was originally developed to measure pain in young children, but it has also been used in elderly with dementia [114]. The psychometric properties of CAS are comparable to those of visual analogue scales [113]. Participants are instructed to rate both the pain intensity and the unpleasantness of the pain from which they suffer at that moment. On the back of the thermometers a value is given to the pain aspects from 0 (no pain/no affect) to 10 (highest pain intensity/highest affect).

#### Faces pain scale (FPS)

The FPS is an instrument to assess the severity of pain on a scale with 7 faces, ranked in order of pain [115]. The participant's score corresponds to the scale number, ranging from 0 (neutral face) to 6 (extreme painful face). It was originally developed to measure pain in young children, but the FPS has also been used in elderly people with dementia [114].

### Statistical analysis

Comparability between the intervention group and the control group will be assessed at baseline to check for differences between the groups on characteristics that may influence the results on the outcome variables. Scores on neuropsychological tests will be converted into z scores and, according to factor analysis, summed up to form specific cognitive domains. A two-way repeated measurement design (T1-T6), with time as within group factor and group (intervention vs control) as between group variable, will be used to analyse the effect of the intervention on the outcome variables.

## Discussion

This paper presents the design of a RCT, which aims to explore the longitudinal effect of regular walking on several health aspects of older people with dementia. The strength of this study is that 1) the intervention is not performed by the research staff, but by the nursing staff which enables it to become a routine in usual care, 2) we have a high number of repeated measurements, i.e. one baseline and 6 post measurements, 3) the various outcome variables enables us to analyse the development of different health aspects within 18 months.

Possible limitations of this study are that 1) only active minded institutions are willing to participate creating a selection bias, 2) there will be a (high) drop-out rate, for example due to death, 3) not all participants will be able to perform/understand all tests.

This method is appropriate to collect data on the effectiveness and feasibility of a walking program. It is also interesting to examine what aspects determine the compliance of the participants. Due to the aging and dementia process, it is not always necessary to increase in test score to find an effect of the intervention, but stabilizing cognitive and behavioural functioning in those who participate in the walking group would also be worthwhile.

## Competing interests

The authors declare that they have no competing interests.

## Authors' contributions

KMV carries out the study and drafted the manuscript. EJAS designed the study and critically revised the manuscript. All authors read and approved the final manuscript.

## Pre-publication history

The pre-publication history for this paper can be accessed here:

http://www.biomedcentral.com/1471-2318/11/38/prepub
